# Coupled Phases and Combinatorial Selection in Fluctuating Hydrothermal Pools: A Scenario to Guide Experimental Approaches to the Origin of Cellular Life

**DOI:** 10.3390/life5010872

**Published:** 2015-03-13

**Authors:** Bruce Damer, David Deamer

**Affiliations:** 1 Department of Biomolecular Engineering; University of California, Santa Cruz, CA 95064, USA; E-Mail: deamer@soe.ucsc.edu; 2 DigitalSpace Research, Boulder Creek, CA 95006, USA

**Keywords:** protocells, hydrothermal fields, early evolution

## Abstract

Hydrothermal fields on the prebiotic Earth are candidate environments for biogenesis. We propose a model in which molecular systems driven by cycles of hydration and dehydration in such sites undergo chemical evolution in dehydrated films on mineral surfaces followed by encapsulation and combinatorial selection in a hydrated bulk phase. The dehydrated phase can consist of concentrated eutectic mixtures or multilamellar liquid crystalline matrices. Both conditions organize and concentrate potential monomers and thereby promote polymerization reactions that are driven by reduced water activity in the dehydrated phase. In the case of multilamellar lipid matrices, polymers that have been synthesized are captured in lipid vesicles upon rehydration to produce a variety of molecular systems. Each vesicle represents a protocell, an “experiment” in a natural version of combinatorial chemistry. Two kinds of selective processes can then occur. The first is a physical process in which relatively stable molecular systems will be preferentially selected. The second is a chemical process in which rare combinations of encapsulated polymers form systems capable of capturing energy and nutrients to undergo growth by catalyzed polymerization. Given continued cycling over extended time spans, such combinatorial processes will give rise to molecular systems having the fundamental properties of life.

## 1. Introduction

The scenario proposed here is based on specific properties of hydrothermal sites characteristic of volcanic regions on today’s Earth that we assume are analogues of similar sites on the prebiotic Earth. Two such sites have been proposed, one associated with submarine hydrothermal activity [1,2] and a second associated with volcanic land-masses emerging through a global ocean [3]. Here, we focus on the second alternative, which we refer to as hydrothermal fields. These are characterized by fluctuating environments in which cycles of hydration and dehydration occur in small ponds undergoing evaporation and replenishment by variable hot springs and precipitation.

Three key features of the model are summarized below:

- Hydration-dehydration (HD) cycles drive molecular systems far from equilibrium
- Lipids encapsulate systems of polymers through multiple cycles, thereby increasing the chance that systems will emerge having one or more functions required for the origin of life.
- Selection of vesicles encapsulating these polymers leads to stepwise increments toward the emergence of functional systems capable of growth, reproduction, and evolution.

Although we believe that the proposed scenario is largely novel, the concept that life began as compartmented molecular systems was suggested earlier by Freeman Dyson, who was quoted recently: “Life began with little bags of garbage, random assortments of molecules doing some crude kind of metabolism … the garbage bags grow and occasionally split in two, and the ones that grow and split fastest win” [4]. This conjecture has been explored in the laboratory with systems of vesicles composed of fatty acids [5,6]. We have also incorporated other conjectures by Vetsigian *et al.* [7] and Doolittle [8] that the first forms of life continuously exchanged genetic information rather than existing as individual organisms. This was recently summarized by Nigel Goldenfeld, who wrote: “Early life was much more collective, much more communal than it is today, particularly the core cellular machinery such as translational machinery … It may well have been that there was massive endosymbiosis, meaning organisms were very porous and could crash into each other and absorb each other on a massive scale and that's how cellular functions were transmitted.” [9] Stuart Kauffman [10] has proposed a theoretical framework that provided us insight on how functional polymers can enable hill climbing through phase spaces of prebiotic evolution toward the origin of life.

## 2. Background

**Figure 1:**
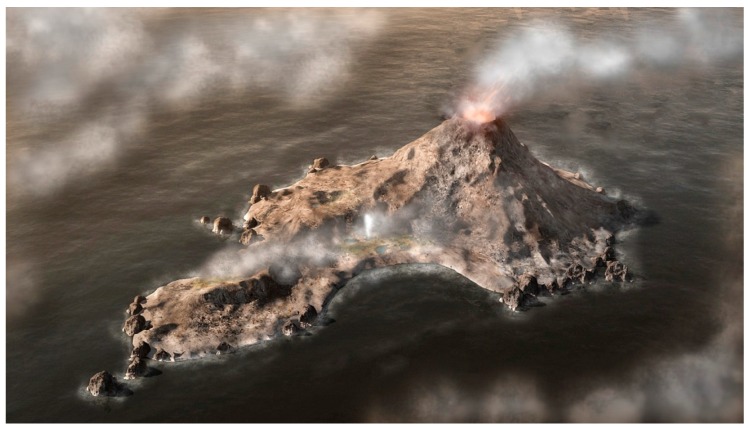
Computer generated conceptual view of a volcanic island rising above the global early Archaean ocean. Precipitation produces small hydrothermal pools that undergo cycles of evaporation and refilling (hydrothermal field and geyser shown at center of island).

**Figure 2:**
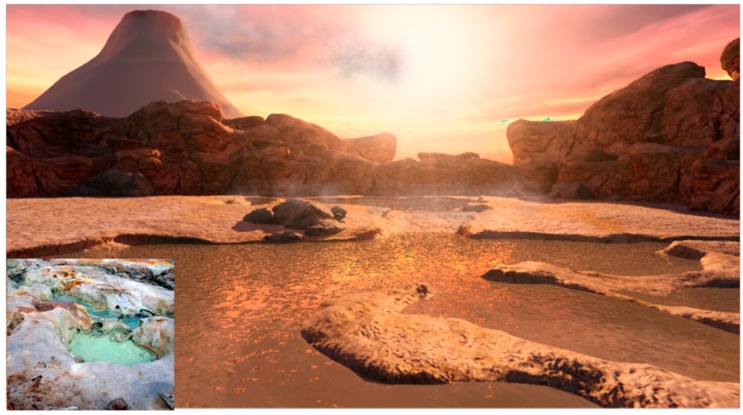
Hydrothermal pools periodically filled by cycles of geyser outflow and rainfall. Mineral surfaces at pool edges provide an environment conducive for concentration of solutes and condensation reactions producing polymers such as oligonucleotides by ester bond synthesis. INSET: Modern analogue: Bumpass Hell, Mount Lassen, California.

**Figure 3:**
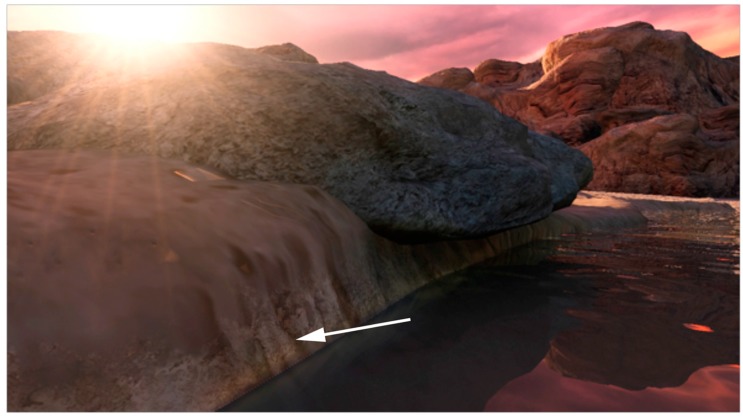
Arrow indicates a film of concentrated dry solutes deposited around edges of fluctuating hydrothermal pools.

The setting for the proposed model is a volcanic island emerging from a global ocean 4 billion years ago (Figure 1 and Figure 2). The atmosphere lacked free oxygen, and precipitation produced small hydrothermal pools fed by hot springs. Similar sites today include geothermal regions of Kamchatka and northern California [11]. During the dehydration phase of a cycle, solutes and amphiphiles accumulate on mineral surfaces to form highly concentrated films (Figure 3). The amphiphiles and minerals provide environments conducive for concentrating solutes and driving condensation reactions.

In such fluctuating environments HD cycles develop deposits of organic solutes on mineral surfaces around the pool (Figure 3). A dynamic cycling between the anhydrous surface phase and the hydrated bulk phase is established as the pool undergoes cycles of evaporation and refilling by precipitation or geyser outflow.

It is highly likely that a variety of organic compounds were present in the prebiotic environment, either synthesized by geochemical processes associated with volcanism or delivered as extraterrestrial infall of comets and meteoritic material. Most of the organics would fall into a global ocean to form an extremely dilute solution. For instance, Stribling and Miller [12] estimated a concentration in the micromolar range depending on assumptions related to the source and sink. However, the organic compounds that fell onto volcanic land-masses or were synthesized by geochemical processes would be dissolved by precipitation and accumulate in pools where they could undergo the reactions proposed here.

We use carbonaceous meteorites as a guide to the kinds of organic compounds that were likely to be available in the prebiotic environment because they were synthesized by non-biological processes in the early solar system. If biologically relevant compounds are detected in a meteorite, it is plausible that similar compounds could be synthesized by geochemical processes on the early Earth. These include potential monomers, such as amino acids and nucleobases, and membrane forming compounds like long chain monocarboxylic acids.

## 3. Dehydration Phase of a Cycle: Assembly of a Multilamellar Matrix

**Figure 4:**
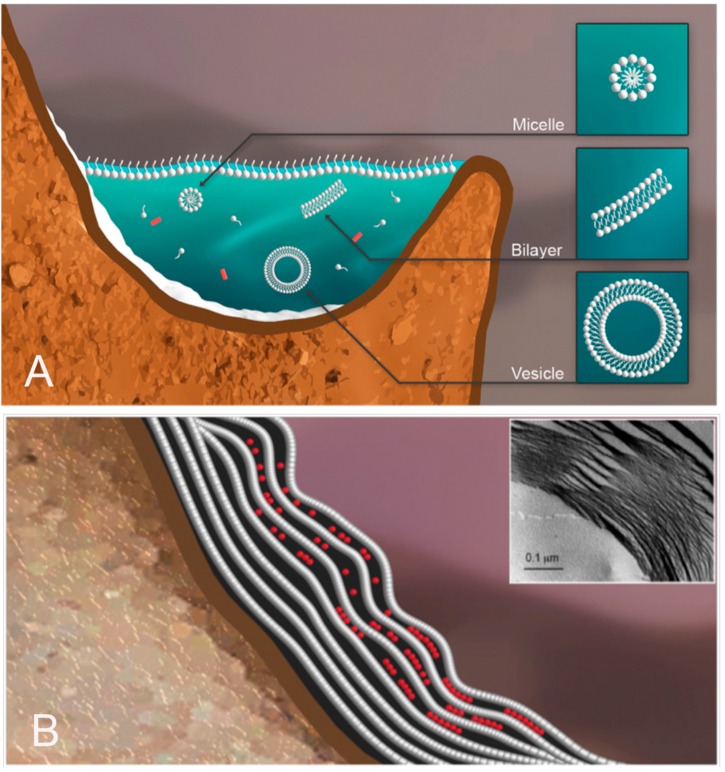
Aqueous phase in a hydrothermal pool. The hydrothermal aqueous phase is a mixture of potential monomers shown in red, and amphiphilic compounds that have assembled into micelles and vesicles (**A**). Upon drying (**B**), the amphiphilic compounds form multilamellar structures with solute molecules organized and concentrated between lamellae [17]. INSET: Freeze fracture image showing a multilamellar matrix of dried phospholipid [18].

To illustrate how HD cycles can promote assembly of vesicles with encapsulated polymers, we will use the example of an aqueous phase consisting of a dilute solution of amphiphilic lipid-like compounds and potential monomers (Figure 4A). The pH is moderately acidic due to dissolved sulfur compounds and carbon dioxide. Rough mineral surfaces create localized sites for solutes and amphiphiles to accumulate during dehydration.

During dehydration, the amphiphiles self-assemble into multilamellar structures that capture concentrated monomers between layers (Figure 4B). Under these conditions, condensation reactions link monomers, such as mononucleotides into polymers [13,14,15,16]. The chemical potential required to drive condensation is made available as water activity is reduced in the anhydrous phase. Under these conditions, water molecules become leaving groups and ester bonds are synthesized. Activation energy is provided by the elevated temperature of the hydrothermal site.

## 4. Hydration Phase: Formation of Vesicles

**Figure 5:**
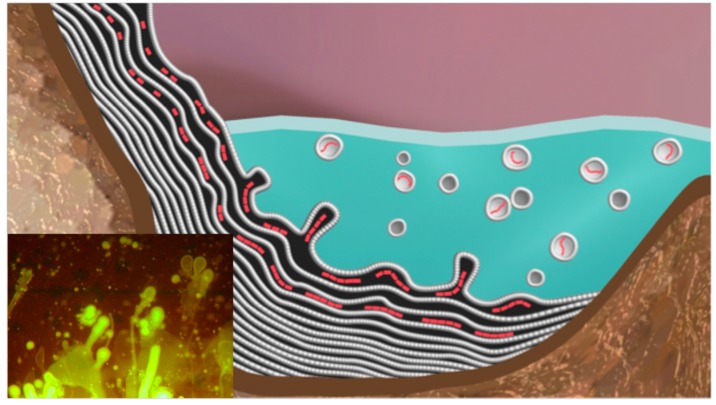
Vesicle formation during hydration phase. INSET: vesicles 10–20 mm in diameter bud from a dried mixture of phospholipid and DNA stained with acridine orange.

In the hydration cycle, vesicles are produced when the water interacts with the dry multilamellar matrix (Figure 5). Some vesicles contain polymers while others are empty [19]. It can be calculated that trillions of vesicles having diameters in the range of typical bacteria are produced from a few milligrams of amphiphiles. Significantly, each vesicle has a different composition from all the rest, allowing a natural version of combinatorial chemistry in which a few vesicles survive while most are dispersed. In regard to the differences that make each vesicle unique, suppose that there are ten different species of amphiphilic components that vary by chain length from 10 to 14 carbons in length, and these have two different head groups, for instance, carboxylate (–COO^−^) and hydroxyl (–OH). The amphipliles form vesicles ranging from 0.5 to 5.0 micrometers in diameter, which are mixed with ten different species of monomers such as amino acids and nucleotides that combine into random sequence polymers having lengths varying from ten to 100 subunits. Simple arithmetic reveals that the number of combinations will be immense. We also note that some of the polymers have the potential to fold into catalytically active conformations, adding another layer of complexity.

## 5. Selection of Vesicles Begins in the Hydrated Phase

**Figure 6:**
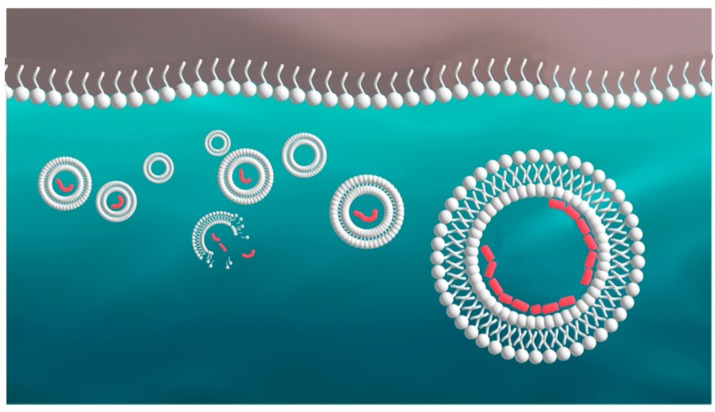
Survival and disruption of protocells. Single-stranded amphiphiles are shown as a monolayer at the atmosphere-water interface and as bilayer vesicles in the aqueous phase. A vesicle may be stabilized if it encapsulates a polymer that interacts with the bilayer surface, as shown in red.

Selection begins during the hydrated phase when some vesicles are lost to the bulk or disrupted while others survive (Figure 6). Survival is promoted by the encapsulated contents of the vesicles. For instance, if a vesicle happens to contain a polymer that stabilizes the membrane, analogous to cytoskeletal proteins of cells today, it will resist disruptive forces such as mechanical shear caused by turbulence.

It is important to note that the amphiphilic components of the system are not pure species, but, instead, include a variety of molecules capable of self-assembly into membranes. These are mixed with monomers and polymers that are not at equilibrium, but in a steady state in which hydrolysis is balanced by synthesis. Furthermore, the mixed amphiphilic compounds are able to associate with the polymers by electrostatic forces, hydrogen bonding and hydrophobic interactions. Because the system is in essence undergoing immense numbers of microscopic experiments during each hydration-dehydration cycle, if specific polymers interact with specific amphiphiles in such a way as to produce a stabilizing effect on the membranes, vesicles composed of those amphiphiles and polymers will undergo selection because the association will persist even during the dehydration phase when vesicle fusion with the surface lamellae occurs. Upon rehydration, the stabilized vesicles will tend to reform, while the components of unstabilized vesicles will be dispersed. Membrane stability, then, is the first selective factor impinging on populations of evolving vesicles, which we refer to as “protocells”.

## 6. Dehydration and Re-Deposition of Polymers to Anhydrous Phase

**Figure 7:**
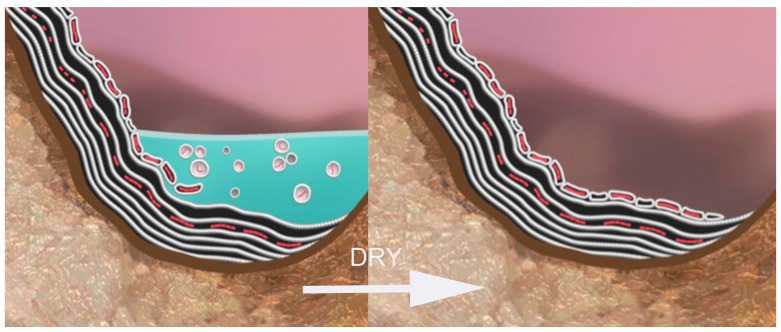
A multilamellar matrix forms again during dehydration.

In the next dehydration cycle (Figure 7), the surviving protocells aggregate on the mineral surface and fuse again with the multilamellar matrix. During this process, the encapsulated contents mix and distribute stabilizing polymers into the next generation of protocells. We term this a *coupling* of the contents of the protocells between the two phases. In other words, two phases of a naturally recurring process—hydration and dehydration—lead to an initial synthesis of a polymer in an anhydrous phase followed by selection of encapsulated polymers in a hydrated phase. The coupled cycles occur indefinitely, thereby allowing accumulation of increasingly complex systems of polymers.

It is interesting to compare hydration-dehydration cycles in fresh water hydrothermal fields to similar cycles that may occur in the sea water of tide pools. The primary ionic constituents of sea water are 550 mM NaCl, 54 mM MgCl_2_, and 10 mM CaCl_2_. In contrast, the typical ionic solutes in hot springs are just a few millimolar in concentration. This means that large and potentially disruptive osmotic gradients would develop across the membranes of vesicles exposed to cycles in a tide pool. Furthermore, divalent cations Ca^++^ and Mg^++^ are known to inhibit self-assembly of amphiphiles such as fatty acids [20]. The osmotic effects would be significantly reduced in a fresh water environment because membranous vesicles exposed to a modest osmotic shock develop transient nanoscopic defects in the bilayers that allow the internal and external solutes to equilibrate, followed by resealing of the bilayer.

## 7. Summary of the Scenario

**Figure 8:**
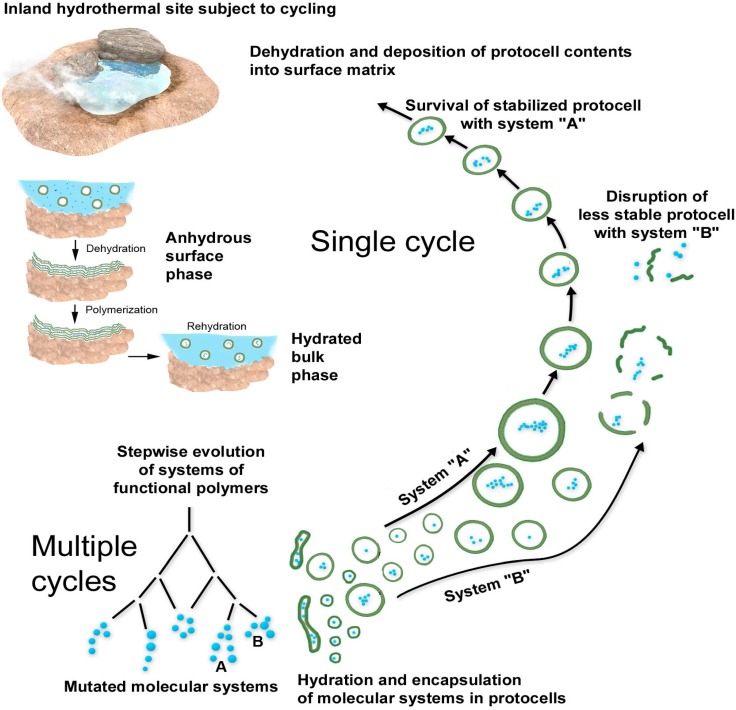
Overview of single and multiple cycles involving coupled phases.

The main features of the scenario are illustrated in Figure 8, which shows the coupled reactions and processing that occurs in hydrated and dehydrated phases. Because the system operates in continuous cycles, reactions do not proceed toward equilibrium but instead undergo a thermodynamic pumping of the system toward a steady state having ever increasing complexity. The polymers accumulate in kinetic traps because the rate of synthesis exceeds the rate of hydrolysis. This is a specific example of the generalized dynamic-kinetic mechanism proposed by Pross [21]. The anhydrous phase could also be thought of as a scaffolding for developing protocells and their functional polymers in the hydrated phase. The anhydrous lamellae concentrate monomers, then organize them in such a way that polymerization is promoted. The resulting molecular systems are tested and selected for functions that enhance the viability of protocells in the dilute bulk phase. The functions of a living cell therefore emerge first by chance, followed by selection and refinement, then gradual incorporation into increasingly functional protocells with each cycle. Early protocells need not rely on the complex and high risk process of replication and division in the bulk phase because the scaffolding of the anhydrous surface phase performs this task.

An important aspect of the scenario is that the polymers are not constant, but instead are in a steady state system in which hydrolysis is balanced by synthesis. Therefore, the system is continuously experimenting, trending toward combinations of polymers that are more stable than other combinations, and polymers that have specific functions, the most important being those that can catalyze their own reproduction.

## 8. Emergence of Functioning Systems Through Coupled Phase Cycles

The next step, although speculative, is one of the most significant features of the scenario that is open to experimental testing. Because the protocells with encapsulated polymers vary in size, composition, and contents, a natural version of combinatorial chemistry becomes possible in which each protocell can be considered to be an experiment. Most will be inert and simply be recycled, but a rare few will happen to have properties that let them survive through multiple cycles. These few protocells constitute a population of ever more complex objects climbing an evolutionary path toward the origin of life. Relatively stable molecular systems within protocells will be preferentially selected [22]. It should be emphasized here that combination and selection, amplification and growth are the sole drivers of this process.

What are the properties of successful protocells? In a sense, the scenario is a version of the “compositional genome” explored by Segre *et al.* [23] in which certain properties of lipid vesicles can be inherited according to their composition rather than information in a polymer sequence. The properties emerge from a synergy between the components of the system, which are the polymers and the surrounding membrane. All of the processes up to this point have been driven by self-assembly and a very simple source of energy—the chemical potential of dehydration that drives ester and peptide bond synthesis so that monomers can form polymers.

Walker *et al.* [24] used computer models to show how sequences of functional polymers can become established in a preexisting pool of non-functional sequences and monomers subject to hydration-dehydration cycles. We develop this further by proposing the mechanism for emergence of specific functional polymers through their cycling within the kinetic trap coupled between anhydrous lamellae and hydrated liposomes. This encapsulated cycling promotes selection between molecular systems at the level of the protocell while reducing losses of products due to diffusion. The evolutionary system that would emerge through such a large number of natural combinatorial experiments could plausibly possess the necessary structures and functions for the transition to cellular life.

## 9. Proposed Polymers

We are not specifying the nature of the polymers, but if the solutes include monomers like nucleotides and amino acids, the polymers would resemble RNA and peptides, as well as possible complexes of RNA and peptides. If so, we can make a list of the functional properties of the polymers, and the list defines the steps required for stepwise evolution of protocells toward living systems.

**S-polymers** have the simplest function, which to bind to and stabilize a membrane-bounded compartment so that its contents are less likely to disperse into the environment. Examples in cells today are cytoskeletal polymers like spectrin that stabilize erythrocyte membranes.

**P-polymers** also have one of the simplest functions, which is to form pores in the bilayer membrane that allow access of potential nutrients to the interior volume. Examples today include short peptides like gramicidin (15mer) and alamethicin (20mer) that form ion-conducting channels in bilayers. Vlassov *et al.* [25] also demonstrated that certain evolved RNA species can also produce defects in lipid bilayers that conduct ions.

**M-polymers** catalyze the steps of a primitive metabolism involving chemical reactions among potential nutrients from the external medium after they enter the protocell. The reactions are a source of energy, and the products can be used for polymerization reactions.

**R-polymers** are able to undergo a primitive version of replication. It may be possible that the monomers not only form polymers under hydrothermal field conditions, but, once formed, can undergo non-enzymatic replication. The reason is that after the first cycle, any newly synthesized polymer can then act as a template. Non-enzymatic template-directed polymerization was first demonstrated by Inoue and Orgel [26] who showed that an activated ribonucleotide monomer such as ImpG can line up on a template composed of oligoC and zip up into a second polymer that has a base sequence complementary to that of the template. This experiment was performed in an aqueous solution, but Olasagasti *et al.* [27] tested whether a similar process might allow non-activated deoxyribonucleotides to assemble on a DNA template and polymerize. All four mononucleotides were mixed with a single stranded DNA 55mer, then exposed to five HD cycles in the presence of phosphatidic acid as an organizing matrix. The double-stranded products were isolated and the synthesized polymer strands were sequenced. It was found that the product strands had sequences matching those of the template with approximately 10% error, a clear indication that template-directed replication can be driven by hydrothermal conditions undergoing hydration-dehydration cycles. A plausible explanation is that both the template strand and monomers become organized in a two dimensional plane when the multilamellar lipid structure assembles during drying. As dehydration occurs, the increasingly concentrated monomers begin to line up on the template by complementary base pairing, and at some point the reduced water activity drives ester bond synthesis that links them into a second strand. More research must be performed to make this a convincing case, but the preliminary indications are promising.

**C-polymers** are a subset of R polymers that happen to be able to catalyze their own replication. An example is the ribozyme polymerase reported by Attwater *et al.* [28].

**F-polymers** are able to provide feedback control for the processes listed above.

**D-polymers** initiate and control the division of a protocell following the duplication of its distinct sets of functional polymers. The first D-polymer may have been a primitive version of FtsZ protein that forms a contractile ring in today's bacteria and is required for cell division [29].

The functions do not need to begin simultaneously. For example, they might emerge as an evolutionary process that involves the following steps:

- An **S-polymer** arises by chance that stabilizes protocell membranes allowing them to survive to return their contents to the anhydrous phase.
- Protocells then evolve the **P-polymer**, which gives them access to nutrients through transmembrane pores.
- Access to nutrients supports the emergence of metabolism catalyzed by **M-polymers**.
- Metabolism will generate products that support replication (**R-polymers**) and systems of molecules that catalyze their own replication (**C-polymers**).
- More robust protocells could then possess molecular systems incorporating feedback loops (**F-polymers**), which control the rates of the above processes.
- **D-polymers** will operate through the collective action of all other functional polymers and enable controlled protocell division in solution. At this point the scaffolding of the anhydrous phase would no longer be needed and, by our definition, the transition to life would occur.

We note that as varieties of functional polymers accumulate in populations of protocells, it also becomes possible for the polymers to interact with one another. For instance, interactions between combinations of oligopeptides and oligonucleotides in protocells may produce novel emergent functions that neither polymer has in isolation. One such emergent function could be a primitive version of coding, a proto-genome, that guides catalyzed polymerization and replication. This possibility suggests two experimental tests (numbers 7 and 8) in section 11 below.

## 10. A Mechanism for Distribution and Increasing Robustness of Early Life

Once life begins it will commence its early existence by consuming local nutrient pools so it must rapidly move to new resources. An advantage to the hydrothermal field setting is that the interconnected pools within the field provide a continuous supply of the building blocks for functional polymers and membranes as well as a dispersal mechanism by which early life can be distributed to new environments. This phase of early life will be characterized by a slow climb to greater functional complexity and robustness through competition and innovation between populations across local ecosystems and changing conditions. One example of increased robustness is that microbial populations originating in a fresh water environment would first need to adapt to brackish conditions of intertidal zones and ultimately be able to thrive in shallow seas.

## 11. Experimental Tests and Predictions of the Coupled Phase Model

The scenario proposed here builds on the results of multiple previous studies, and it is useful to summarize this weight of evidence before proposing new experiments:

- All of the primary species of organic compounds that are required—amino acids, nucleobases, and monocarboxylic acids – are present in carbonaceous meteorites. This fact makes it plausible that they were likely to be available in the prebiotic environment, either delivered during late accretion or synthesized geochemically [30,31,32].
- A variety of amphiphilic compounds form membranous vesicles by self-assembly. Some of these are as simple as fatty acids, also present in carbonaceous meteorites [33].
- Cycles of hydration and dehydration are ubiquitous in hydrothermal fields.
- Upon drying, amphiphilic compounds fuse into multilamellar structures that capture and concentrate monomers between bilayers [17].
- Water activity is reduced during dehydration to the point that condensation reactions link the concentrated monomers into polymers [13,15,16].
- Polymers are readily encapsulated in membranous vesicles by HD cycles [19].

The following experiments will test the validity of the model:

(1) It should be possible to demonstrate that distinct molecular systems of encapsulated polymers increasingly tend to persist when lipid vesicles are cycled multiple times between the anhydrous lamellar phase and the hydrated protocell phase.
(2) If monomers are present, they will form polymers having random sequences of subunits, but the sequences will become increasingly specific over time as selection occurs.
(3) When a selective hurdle, such as vesicle survival, is imposed during cycling, an S-polymer will emerge which enhances membrane stability.
(4) The S-polymer may not only stabilize vesicles, but its interaction with lipid could also stabilize the polymer against hydrolysis. This produces a positive feedback loop so that the polymer accumulates during cycling until it dominates the composition of vesicles.
(5) If the imposed selective hurdle is related to permeability, a P-polymer will emerge which allows polar or ionized nutrient solutes to cross the membrane barrier.
(6) Indirect and direct competition between protocells will become apparent in later cycles.
(7) Polymers will begin to exhibit catalytic properties over time, and at some point a polymerase will emerge that catalyzes replication.
(8) The polymerase activity could be enhanced by the interaction between two different polymer species such as ribozymes and oligopeptides. Such interactions are likely to be a fruitful topic of future research.
(9) Populations of functional protocells will become increasingly homogeneous in later cycles as one population with a specific composition is better able to survive selective hurdles or more efficiently uses available resources.

## 12. Discussion and Conclusions

We have argued here that plausible geological sites for selective processes are fluctuating hydrothermal pools associated with volcanic land-masses scattered over the surface of the early Earth. Based on observations of hydrothermal locales in prebiotic analogues, such as Kamchatka, Iceland, Yellowstone, and Hawaii, there are three general types. Simplest is the Hawaiian version composed of lava surfaces and ash, with little or no silicate sinter or clay deposits. Precipitation travels downhill into the pools and variations in rainfall lead to hydration-dehydration cycles as pools fill and evaporate. The water is very low in ionic content, with solutes at millimolar concentrations, and the insoluble basaltic surfaces are equivalent to laboratory glassware.

The second version is represented by the clay-lined ponds present in Kamchatka and Iceland. The clays are formed by hydrothermal processing as acidic water interacts with volcanic minerals over long time intervals. The clay surfaces strongly adsorb ionic and polar solutes, but not amphiphilic compounds that can assemble into membranous structures (11). The third version is ponds like those in Yellowstone, in which silicate sinters and carbonate minerals accumulate around the rims. We assume that if any of these fluctuating hydrothermal field conditions were present in the prebiotic Earth, organic solutes would accumulate in the pools and form films on mineral surfaces as they undergo cycles of hydration and dehydration.

Two concerns should now be addressed that are specifically related to this scenario: What is the source of potential monomers in such sites, and how might they avoid damage by the more intense ultraviolet radiation of the early Earth? 

To answer the first question, we note that the primary sources of organic compounds on the prebiotic Earth are still being debated. Two obvious possibilities, not mutually exclusive, include the continuous influx of organic material on interplanetary dust particles that is still occurring today [34], synthesis in the atmosphere [35] or volcanic geochemical processes [36]. Given these potential inputs, it is reasonable to assume that the mineral surfaces of volcanic land-masses would accumulate thin deposits of organic compounds from one or both of the above sources. Potential monomers in the mixtures would be transported to hydrothermal ponds by precipitation and accumulate there. Regarding degradation by ultraviolet radiation, this concern arises from the sterilizing effect of UV light on microbial life today. However, there have been no studies of potential damaging effects of UV light on mixtures of organic compounds in anaerobic hydrothermal ponds, so this is a moot point. We also note that the processes we described do not require light energy, but could equally well occur in shaded areas.

The scenario we proposed here is based on the statistics of large numbers. Assuming that random polymers can be synthesized and then encapsulated as protocells, it follows that a near-infinite number of combinations will occur. These represent “experiments” that can be subjected to selection and evolution, but the process cannot take place under purely aqueous conditions. Instead the environment must provide cycles in which polymerization of monomers occurs in one phase of the cycle, followed by dispersal of encapsulated molecular systems in another phase. Only then can populations of protocells emerge that are capable of selection and evolution toward the first forms of cellular life.

In an earlier demonstration of the power of large numbers, Bartel and Szostak [37] exposed random sequence RNA to cycles of selection and amplification, with the selection target being ligase activity. Although only a few molecules in trillions of random RNA sequences had ligase activity at the start of the experiment, after ten cycles of selection and amplification those few evolved into the dominant species of RNA present, with a seven million-fold increment in ligase activity. We have expanded this model to conditions that were likely to be common on the early Earth and still exist today. Cycles of hydration and dehydration in hydrothermal pools, coupled with a process that produces encapsulated polymers, constitute a ‘combinatorial engine’ with the power to explore vast numbers of combinations. At its core, the model proposes a natural scaffolding, incorporating physical processes that support the emergence and selection of functions through encapsulated systems of catalysts and polymers that then become capable of encoding heritable traits. The transition to life occurs when these encapsulated systems of polymers work in concert to support growth and propagation of protocells entirely in the aqueous phase, with the cycling through the scaffolding of the anhydrous phase no longer being required.
